# A commentary on ‘Personal versus therapist perioperative music intervention: a randomized controlled trial’

**DOI:** 10.1097/JS9.0000000000001507

**Published:** 2024-05-03

**Authors:** Biao Liang, Yiheng Yang, Zhenyi Wang

**Affiliations:** Department of Anorectal Surgery, Yueyang Hospital of Integrated Traditional Chinese and Western Medicine, Shanghai University of Traditional Chinese Medicine, Shanghai, People’s Republic of China

*Dear Editor,*

We read with great interest the recent article ‘Personal versus therapist perioperative music intervention: a randomized controlled trial’ published in the *International Journal of Surgery* ^1^. This study was a prospective, parallel-group, single-blinded, randomized controlled study that evaluated whether personal music intervention with patient-selected songs played ad libitum is more effective than standard therapist-designed treatment with classical music. Jiang *et al*. found that personal music intervention improved postoperative systolic blood pressure, anxiety, nausea, emesis, and overall satisfaction, as compared to therapist-designed classic intervention. This study stood out as the first to focus on the relationship between postoperative music personal preferences and efficacy. These findings are both timely and extremely significant. While expressing deep appreciation for the rigorous efforts and valuable contributions of this study, there are also some constructive suggestions for further improvement.

First, regarding the study design, although the authors have set strict parameters for participant exclusion, we propose the addition of the following exclusion criteria: ‘1. History of severe insomnia’ and ‘2. Presence of obvious psychiatric symptoms such as anxiety and depression.’ These considerations are essential to further improve the reliability and rigor of the findings, as they may influence the assessment of postoperative anxiety and sleep quality^2^.

Second, the clinical endpoints of this study included postoperative nausea and vomiting (PONV) during the 24 h following surgery. Undeniably, postoperative nausea or vomiting is also considered a complication of anesthesia^3^. It is essential to take into account that different anesthetists may have different operating practices in anesthetic interventions. However, this study did not report the details of the procedure. Therefore, we recommend the use of the same anesthetist involved in surgery to reduce the bias associated with anesthesia.

Finally, the study used a single-center study, which may introduce some study bias^4^. Large multicenter studies are required to validate this finding. Also, this study’s participants were involved in various types of surgery. However, there are variations in the surgical approach of different surgical teams and levels of skill, which will have a greater impact on the patient’s postoperative recovery, thus affecting the results of the study^5^.

In conclusion, the study by Jiang *et al*. has taken an important step forward in our understanding of the relationship between postoperative music personal preferences and efficacy. The convoluted relationship between postoperative music personal preferences and efficacy emphasizes the significance and necessity of further research in the surgical field. We appreciate the opportunity to provide recommendations on this study and look forward to more innovative works from the authors in the future and expect our recommendations to contribute to its continued academic excellence.

## Ethical approval

Not applicable.

## Sources of funding

This study was supported by the National Natural Science Foundation of China (No. 82274531).

## Author contribution

B.L., Y.Y., and Z.W.: designed and wrote this letter.

## Conflicts of interest disclosure

The authors declare no conflicts of interest.

## Research registration unique identifying number (UIN)

Not applicable.

## Guarantor

Zhenyi Wang.

## Data availability statement

This manuscript is a correspondence. No data were generated during the writing of this study.
